# Phase Reconstruction of Low-Energy Electron Holograms of Individual Proteins

**DOI:** 10.1021/acsnano.2c06897

**Published:** 2022-11-11

**Authors:** Hannah Ochner, Sven Szilagyi, Moritz Edte, Luigi Malavolti, Stephan Rauschenbach, Klaus Kern

**Affiliations:** † Max Planck Institute for Solid State Research, Heisenbergstrasse 1, DE-70569 Stuttgart, Germany; ‡ Department of Chemistry, University of Oxford, 12 Mansfield Road, Oxford OX1 3TA, U.K.; § Institut de Physique, École Polytechnique Fédérale de Lausanne, 1015 Lausanne, Switzerland

**Keywords:** low-energy electron holography, hologram reconstruction, phase retrieval, single-molecule imaging, protein imaging

## Abstract

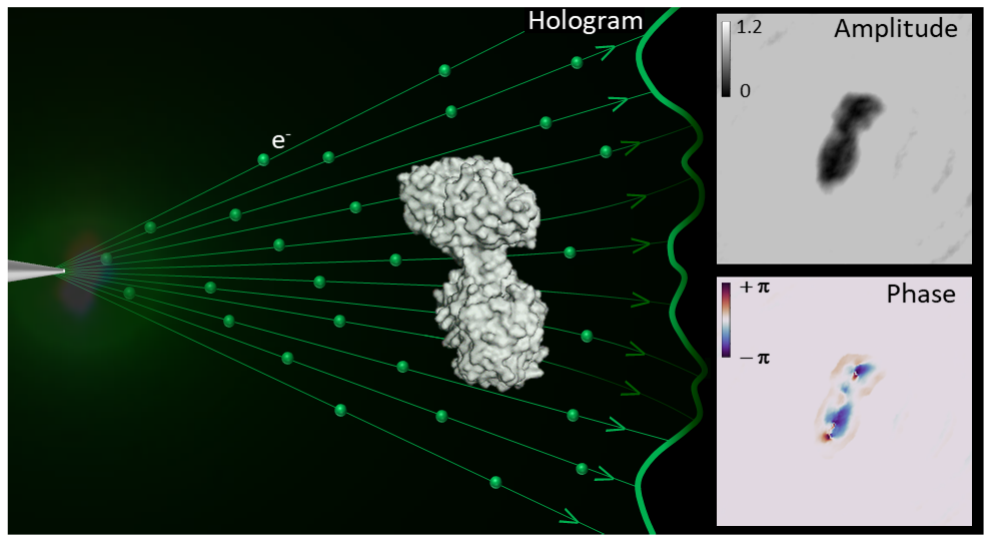

Low-energy electron holography (LEEH) is one of the few techniques capable of imaging large and complex three-dimensional molecules, such as proteins, on the single-molecule level at subnanometer resolution. During the imaging process, the structural information about the object is recorded both in the amplitude and in the phase of the hologram. In low-energy electron holography imaging of proteins, the object’s amplitude distribution, which directly reveals molecular size and shape on the single-molecule level, can be retrieved via a one-step reconstruction process. However, such a one-step reconstruction routine cannot directly recover the phase information encoded in the hologram. In order to extract the full information about the imaged molecules, we thus implemented an iterative phase retrieval algorithm and applied it to experimentally acquired low-energy electron holograms, reconstructing the phase shift induced by the protein along with the amplitude data. We show that phase imaging can map the projected atomic density of the molecule given by the number of atoms in the electron path. This directly implies a correlation between reconstructed phase shift and projected mean inner potential of the molecule, and thus a sensitivity to local changes in potential, an interpretation that is further substantiated by the strong phase signatures induced by localized charges.

In-line low-energy electron holography (LEEH)^1^ has been shown to be capable of nondestructive imaging of biomolecules^2^ at the single-molecule level at subnanometer resolution.^3,4^ Due to the high contrast obtained by employing low-energy electrons, LEEH imaging can forego the averaging step that is central to well-established high-resolution structure determination methods for proteins such as cryo electron microscopy (cryo-EM) and X-ray crystallography.^5−7^ LEEH, as an emerging imaging method, could thus serve as a complementary tool to these techniques as it allows for the imaging of classes of molecules that are difficult to image with averaging methods,^8,9^ such as molecules with a high degree of conformational variability.^3^

In LEEH imaging, a coherent beam of low-energy electrons (50–200 eV) is used to generate the holographic image of individual molecules. The information about the object is stored in both the amplitude and the phase of the complex-valued wave field Ψ_O_ resulting from the interaction of the electron beam with the object, whose interference with the unperturbed reference wave Ψ_R_ yields the superposition *U* = Ψ_O_ + Ψ_R_, which in turn generates the hologram *H* = |Ψ_O_ + Ψ_R_|^2^ in the detector plane (Figure 1a).^10−12^ Holography is thus not a real-space imaging method, and the imaging process consists of two steps: the experimental acquisition of a hologram, during which the structural information about the imaged object is recorded in the form of an electron hologram,^10,11,13^ and the subsequent numerical image reconstruction.^12,14^

**Figure 1:**
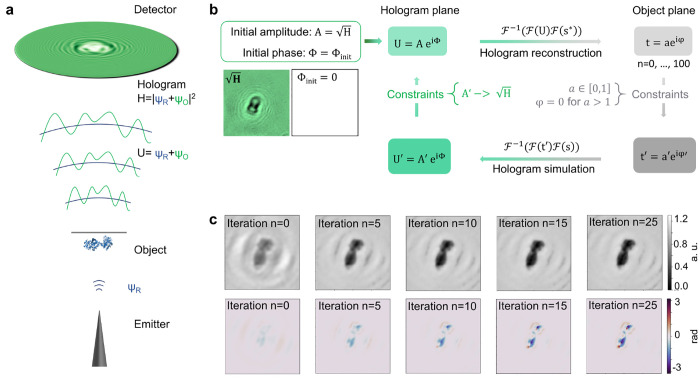
Hologram generation and iterative hologram reconstruction algorithm. (a) Sketch of the LEEH setup, consisting of an electron emitter, a protein sample deposited on free-standing single-layer graphene (the object) and a detector to record the hologram. The hologram (*H*) is generated as the interference pattern between the wave scattered by the object (Ψ_O_) and the unscattered incident reference wave (Ψ_R_). (b) Schematic representation of the iterative reconstruction algorithm used for the reconstructing amplitude and phase images of the object. During the reconstruction process, a complex wave field is propagated between the hologram plane and the object plane using a numerical implementation of a Fresnel–Kirchhoff integral. In both planes, a separate set of constraints is applied in each iteration step. (c) Amplitude (top) and phase (bottom) reconstructions after *n* = 0, 5, 10, 15, and 25 iteration steps. All images are scaled to the same value range as indicated by the color bars. In this example, convergence is reached after approximately 25 iterations. The elimination of the fringe pattern in the background of the reconstructed images with an increasing number of iterations demonstrates that the iterative process removes the contributions originating from the twin image.

Up to now, the focus of LEEH studies has been on the structural information obtainable from the amplitude data encoded in a hologram,^3,4^ which can be reconstructed with the help of a one-step propagation-based algorithm.^3,4,12,15^ The reconstructed amplitude maps the object’s interaction with the incident electrons in the form of inelastic scattering events. This interaction can yield a reduction of the amplitude of the resulting wavefront in the hologram plane by electron absorption, high-angle scattering, and a loss of coherence with respect to the incident reference wave due to the energy transfer during inelastic scattering events.^16−18^ Since inelastic events play a significant role in low-energy electron scattering due to the high electron scattering cross sections in this energy range,^19−21^ the amplitude reconstructions are very well suited for the characterization of molecular shapes and sizes.^3,4^

In general, however, the interaction of low-energy electrons with biological matter involves both elastic and inelastic scattering processes, resulting in a change of both amplitude and phase of the incident wave.^17,19,20^ Changes in electrostatic potential primarily induce elastic scattering events, which are characterized by the conservation of kinetic energy and an altered propagation direction, resulting in a path difference and thus a phase shift with respect to the incident wave.^16,17^ Retrieving the phase shift induced by the presence of the object could thus allow the mapping of the object’s mean inner potential,^16,22^ defined as the spatially averaged electrostatic potential of the object,^19^ as well as the observation of localized electric fields, such as those related to the existence of local charges.^23^

Unlike amplitude information, however, LEEH phase information cannot directly be obtained via a one-step reconstruction algorithm since only relative phase information is retained in the hologram,^10,11,14,24^ while the absolute phase in the detector plane is lost. This lack of absolute phase information in the detector plane, which is common to many imaging techniques, is often referred to as the phase problem.^25−27^ In LEEH imaging, phase thus has to be retrieved by other means, for instance, iteratively, for which the algorithm proposed by Latychevskaia and Fink^14^ can be employed. This phase retrieval scheme is based on the iterative routine suggested by Gerchberg and Saxton,^24^ which is well-established in phase retrieval for many different imaging techniques.^28−32^ The algorithm involves a stepwise hologram reconstruction-simulation process imposing constraints in both the hologram plane and the object plane and has been successfully applied to simulated hologram data^14^ as well as to experimental holograms of charged impurities on graphene.^23^ Despite these promising results, an application of the phase retrieval method to complex molecular systems has not been reported yet, and as such, an interpretation of the information encoded in the LEEH phase signal of such objects is still lacking.

In this paper, phase reconstructions of LEEH holograms of individual proteins are obtained by applying the aforementioned phase retrieval scheme,^14^ augmented by an additional phase constraint, to the experimental data. After presenting the algorithm, with particular emphasis on the discussion of the constraints enforced in each iterative step, the iterative phase and amplitude reconstructions of experimentally acquired protein holograms are examined. The results demonstrate that, among additional contributions to the phase, there is a strong correlation between the measured phase distribution and the number of atoms in the electron path, which can be established by comparing the reconstructed phase shift with the projected atomic density of molecular models of the imaged proteins. Since proteins mainly consist of light atoms with similar scattering strengths in the relevant energy range, the observed correlation between phase shift and projected atomic density implies that the reconstructed phase shift maps changes in the mean inner potential of the molecule, which, at the low electron energies employed by LEEH, is mainly probed via electron–electron scattering. The connection between induced phase shift and local electrostatic potential is further affirmed by the sensitivity of the measured phase distribution to localized charged features.

## Iterative Reconstruction Algorithm

The lensless in-line LEEH setup discussed here consists of an electron source in the form of a sharp tungsten tip, which field-emits electrons in an energy range of 50–200 eV, the protein sample deposited by native electrospray ion beam deposition (native ES-IBD)^33−35^ onto a free-standing single layer graphene (SLG) substrate^36^ (see Methods section), and a microchannel plate detector to record the hologram, which is photographed with a digital camera and subsequently numerically reconstructed (Figure 1a).

The reconstruction process for in-line holograms, which has been shown to yield amplitude reconstructions of proteins in a one-step reconstruction routine^3,4,12^ and is implemented in each step of an iterative phase retrieval scheme, is based on wave field propagation between the object plane and the detector plane.

This propagation step is described by a Fresnel–Kirchhoff integral, which takes the form^12,37^
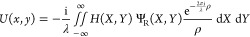
1with 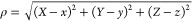
*U*(*x*, *y*) is the reconstructed exit wave in the object plane, *H*(*X*, *Y*) is the measured hologram, Ψ_R_ is the reference wave, *z* is the source-to-sample distance, and λ is the electron wavelength. (*x*, *y*, *z*) are the coordinates in the object plane, while (*X*, *Y*, *Z*) denote the coordinates in the detector plane.

Since the integral has the form of a convolution, the convolution theorem can be used to express the exit wave as a series of Fourier transforms^12^
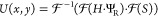
2with the propagator function .

With this propagation step, an iterative algorithm can be outlined (see Figure 1b), which removes the unphysical contributions to the complex wave field caused by the loss of absolute phase information at the detector and thus allows for an accurate reconstruction of the phase distribution of the object.^14^ The propagation step connects the complex wave field *U*(*X*, *Y*) in the detector plane with the transmission function *t*(*x*, *y*) = *a*(*x*, *y*) exp(iφ(*x*, *y*)) in the object plane, which describes the absorbing and phase-shifting properties of the object.^14^ In each iterative step, a complex-valued wave field, created from the measured hologram and an initial random phase distribution, is propagated between the hologram plane and the object plane with constraints being applied in both planes.^14^

In Figure 1c, a series of reconstructed amplitude and phase distributions of an experimentally acquired LEEH hologram of a transferrin molecule is shown after different numbers of iterations. In this example, both amplitude and phase converge to a stable output after approximately 25 iterations; i.e., the mean squared error between the images reconstructed in subsequent iterations^14^ is below a threshold value of 10^–5^ (see Figure S1 for further details). Since the reconstructions of experimental holograms measured on our setup usually converge after 50 iterations or less, we use 100 iterations as a default value to have a standard for comparison certain of convergence.

This type of iterative reconstruction scheme exhibits a high degree of flexibility regarding possible constraints.^14,38^ In general, the choice of constraint depends on the imaging situation as well as on prior knowledge about the imaged object. In LEEH, the measured data set is acquired in the hologram plane, hence the constraint to be enforced in this plane is straightforward to choose: the amplitude distribution *A*′ calculated in the hologram plane in each iterative step must be replaced by the measured amplitude distribution *A* = . Since the measurement does not yield direct information about the object plane, a generally applicable constraint needs to be chosen that does not require prior knowledge about the object and does not risk the implementation of a bias toward certain results. One possibility is to turn the requirement that energy needs to be conserved during the imaging process into a constraint, as suggested in Latychevskaia et al.^14^ An interaction of the incident wave with the object will, in general, lead to reduced transmission but can never lead to increased transmission. This, in turn, means that the amplitude of the exit wave must be lower or equal to the amplitude of the incident wave, *a* ≤ 1. Given the relation between amplitude and absorption, *a* = exp(−α), this is equivalent to requiring the object’s absorption distribution to be non-negative, α ≥ 0.^14^ In the numerical implementation, amplitude values of pixels that violate this requirement are thus set to 1.

**Figure 2:**
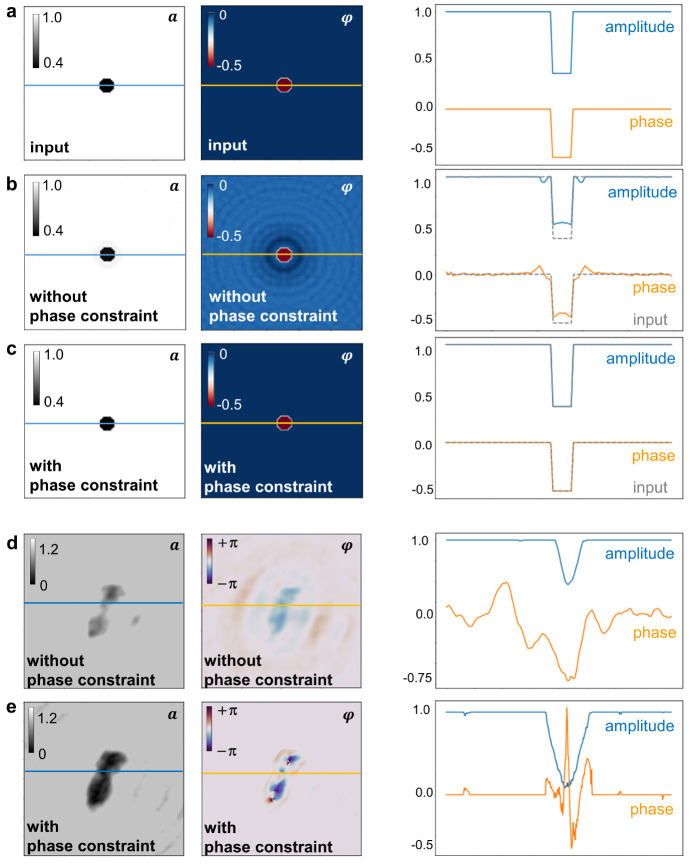
Comparison of iterative phase retrieval with and without phase constraint. (a–c) Simulated example. (a) Left to right: input amplitude and phase distributions, along with the corresponding cross sections along the lines indicated in the images, used to simulate the hologram from which the reconstructions in (b) and (c) are obtained. (b) Left to right: Reconstructed amplitude and phase distributions resulting from an iterative phase retrieval algorithm enforcing only the amplitude constraint, but not the phase constraint in the object plane. The corresponding cross sections are depicted as solid lines; for comparison, the cross sections through the input are added as gray dashed lines. (c) Left to right: Reconstructed amplitude and phase distribution resulting from the iterative routine employing both the amplitude and the phase constraint in the object plane. The input (a) is perfectly reconstructed as demonstrated by the comparison of both the images and the cross sections. (d, e) Experimental example. (d) Left to right: Amplitude and phase reconstruction and corresponding cross sections of an experimentally acquired hologram of a transferrin molecule reconstructed without phase constraint. (e) Left to right: Amplitude and phase reconstruction and corresponding cross sections of the same hologram as in (d) reconstructed with both amplitude and phase constraint.

For these constraints to be effective, the hologram is normalized by division by the background intensity such that the resulting background amplitude is set to unity. While it is possible to only constrain the amplitude in the object plane and leave the phase unaltered,^14,23^ an efficient and robust reconstruction of both the amplitude and phase distributions additionally requires the phase to be constrained in the object plane (see Figure 2, Figure S2, and Figure S3). The constraint on the phase enforced here involves setting the phase value to zero at pixels where the amplitude exceeds the value 1. Such high amplitude values are generated by contributions from the twin image,^11,13,14^ an artifact occurring in the reconstruction of in-line holograms due to the loss of absolute phase in the detector plane, which most prominently appear in the form of fringes in the background of the image.^14^ Since the relative phase shift in the background is assumed to be constant and equal to zero (see Methods), constraining the phase by setting the phase values of pixels with amplitudes larger than 1 to 0 promotes the recovery of a uniform background phase. With this additional constraint on the phase, the iterative process perfectly reconstructs (Figure 2c) the input amplitude and phase distributions of a simulated object (Figure 2a), a feat that is not accomplished by the iterative process that enforces only the amplitude constraint (Figure 2b). This can be explained by the fact that the contributions originating from the twin image in the object plane generate both pixels of negative absorption (amplitude >1) and pixels of positive absorption (amplitude <1) in the image background. In each iteration, the negative absorption values are partially corrected by the amplitude constraint, while the positive absorption pixels are left unchanged. The amplitude constraint can thus adequately remove the negative absorption artifact but does not affect the positive absorption counterpart, which especially has an adverse effect on the reconstructed phase values (see Figure S2e and Figure S3a,c). The additional constraint on the phase, although still limited to the negative absorption pixels, affects the phase estimate for the neighboring pixels and thus leads to a stepwise correction of both absorption and phase values over the whole of the image (see Figure S2f and Figure S3b,d).

**Figure 3:**
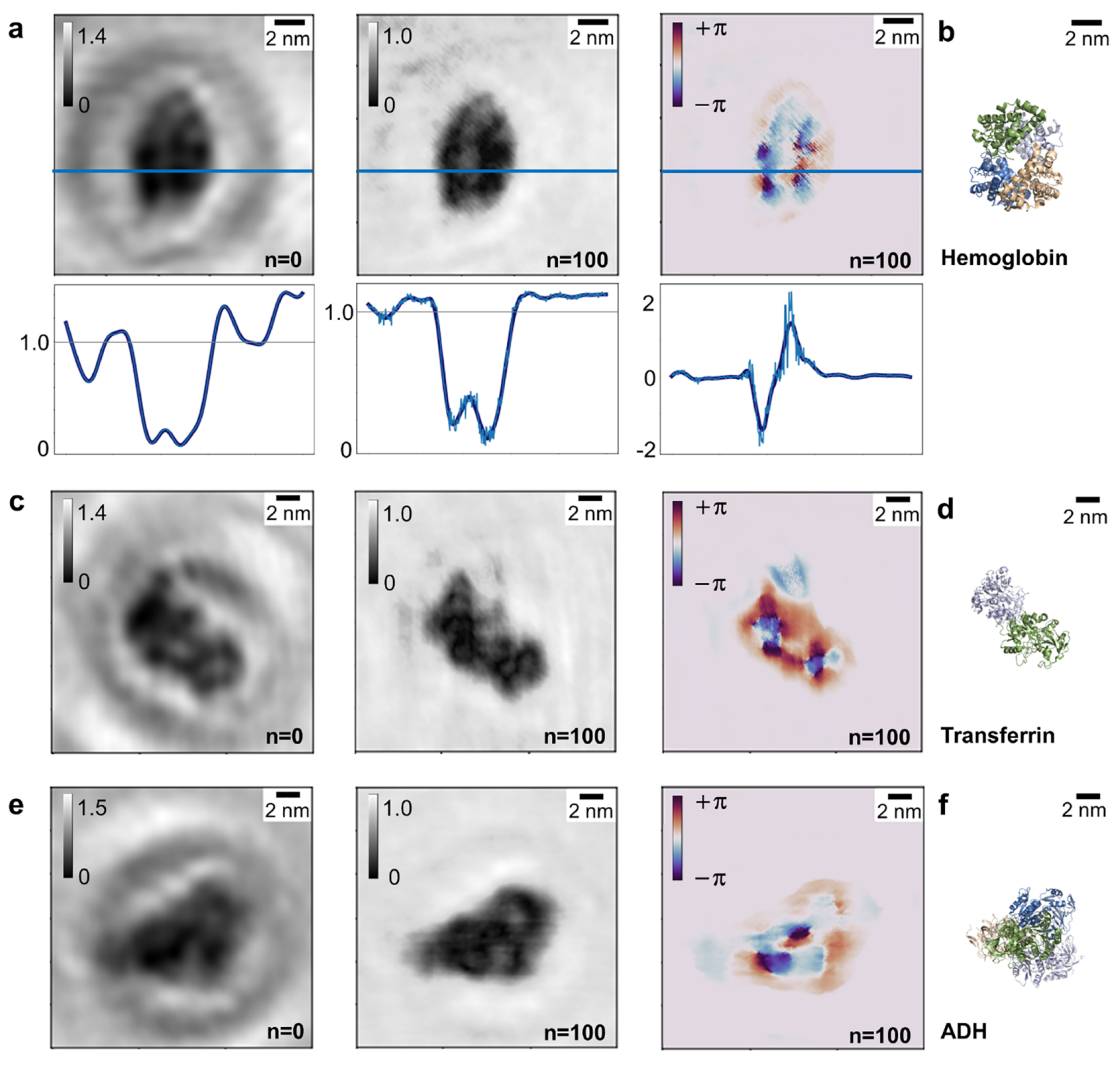
Amplitude and phase reconstructions of protein holograms. (a) Left to right: one-step amplitude reconstruction, iterated amplitude reconstruction after 100 iterations, and phase reconstruction after 100 iterations of a hemoglobin molecule and cross sections along the blue lines indicated in the images. The cross sections (light blue) have been smoothed with a Savitzky–Golay filter (dark blue) for enhanced clarity. (b) Crystallographic model of a hemoglobin molecule (PDB: 1FSX(39)) in an orientation matching the one observed in (a). (c) Left to right: one-step amplitude reconstruction, iterated amplitude reconstruction after 100 iterations, and phase reconstruction after 100 iterations of a transferrin molecule. (d) Crystallographic model of a transferrin molecule (PDB: 1JNF(40)) in an orientation matching the one observed in (c). (e) Left to right: one-step amplitude reconstruction, iterated amplitude reconstruction after 100 iterations and phase reconstruction after 100 iterations of an ADH molecule. (f) Crystallographic model of an ADH molecule (PDB: 7KCQ(41)) in an orientation matching the one observed in (e).

Enforcing the phase constraint also increases the algorithm’s robustness regarding both random phase inputs as well as high absorption values (see Figure S2 and Figure S3). The latter is of particular relevance in the case of experimental data since high absorption values frequently occur in this context (see for example amplitude reconstructions in Figure 3), while the former ensures a convergence to the same output distribution independently of the phase input. In the experimental case (Figure 2d,e), a direct comparison to the input is not possible, but the reconstruction with phase constraint (Figure 2e) shows a clear improvement when compared to the reconstruction without phase constraint (Figure 2d). Without phase constraint, contributions due to the twin image cannot be efficiently removed in the iterative reconstruction, as can be seen by the presence of the strong fringe pattern in the background of the phase reconstruction, visible in both the image and the corresponding cross section (Figure 2d). Thus, in the following, the iterative reconstructions are obtained by applying both the amplitude and the phase constraint in the object plane.

## Results

The application of the iterative reconstruction scheme to simulated data demonstrates that the correct input amplitude and phase distributions can be retrieved (Figure 2a–c, Figure S2, and Figure S3), and the example of the transferrin molecule depicted in Figures 1 and 2 shows that the algorithm can also be applied to experimentally acquired LEEH holograms. Figure 3 shows several examples of one-step (*n* = 0, left panels) and iterative (*n* = 100, central and right panels) reconstructions of experimental holograms of different proteins. In order to ensure convergence, *n* = 100 iterations has been chosen as a default value (see Supporting Information). Since the propagator function used to obtain the one-step amplitude reconstructions is also employed in each step of the iterative reconstruction scheme, the object plane output of the zeroth iteration (*n* = 0) corresponds to the one-step reconstruction of the image. While the one-step amplitude reconstructions provide an accurate estimate of the size and shape of the molecules^3,4^ (Figure 3), strong intensity modulations are observable in the background, which can be attributed to contributions from the twin image. The comparison between one-step and iterative reconstructions shows that the contributions from the twin image are effectively suppressed in both iterated amplitude and phase, as demonstrated by the strong reduction of the fringes in the background of the images.

For reconstruction, the holograms were normalized by division of the hologram by its mean intensity, resulting in a mean background amplitude of 1 (arbitrary units) and a range of reconstructed amplitude values that extend from 0 to slightly above 1. The reconstructed phase, on the other hand, takes both positive and negative values in the range of [−π, +π) rad. Since, at the energy range employed in LEEH, the observed phase shifts could originate from a variety of interactions ranging from electrostatic interaction via polarization effects to local electric fields due to the charge distribution within the molecule, the interpretation of the simultaneous occurrence of positive and negative phase shifts in the 2π-periodic direct output of the algorithm is complex. On the one hand, a change in sign could be a direct result of this periodicity, manifesting in the occurrence of phase wrapping when the measured phase shift exceeds the [−π, +π) rad value range. On the other hand, it is conceivable that due to sign changes of the local potential, both positive and negative phase shifts could occur simultaneously, for example, if local accumulations of negative and positive charge are present in the same image. Changes of the phase sign originating from either of these effects could in principle occur within the same reconstruction. While the phase sign changes due to phase wrapping could be accounted for by employing phase unwrapping methods, it is not straightforward to choose a suitable phase unwrapping algorithm in the case of LEEH since different algorithms applied to experimental LEEH phase data yield different results (discussed in detail in the Supporting Information). Given this ambiguity in phase unwrapping as well as the fact that a full description of the interaction of low-energy electrons with the sample is not available at present and a study of it would be beyond the scope of this manuscript, here we chose to use the direct output of the phase retrieval algorithm as the basis for the discussion of the interpretation of LEEH phase data.

In all cases, the amplitude reconstructions succeed in retrieving the molecules’ shapes and sizes, as can be confirmed when comparing the reconstructed images to the respective molecular models, as shown in Figure 3b,d,f, ascertaining the orientation of the imaged proteins with respect to the surface as well as the structural intactness of the molecules.

While the phase reconstruction maps size and shape as well, albeit with less sharp edges, it provides additional insight into the molecules’ inner structure, as will be discussed in the following using the examples of the three different proteins shown in Figure 3.

In the case of the hemoglobin molecule shown in Figure 3a, the four subunits as well as the cavity in the center of the molecule are recognizable to varying degrees in the reconstructed images. While the subunits of the molecule (Figure 3b, each subunit is depicted in a different color) are difficult to distinguish in the one-step reconstruction (left) and the cavity in the center of the molecule is barely visible, the contrast within the molecule is enhanced by the iterative process, which becomes apparent when comparing the cross sections of the reconstructions shown in Figure 3a. The highest contrast within the molecule is observed in the phase reconstruction, where the four subunits as well as the central cavity are clearly resolved. The cavity in the center of the molecule exhibits the same phase shift as the background, as demonstrated by the corresponding cross section. This suggests that the magnitude of the reconstructed phase shift is sensitive to the variations in the thickness of the molecule and hence to changes in the number of atoms in the electron path.

The phase map of the transferrin molecule depicted in Figure 3c shows two prominent phase features, corresponding to negative phase values (blue) within the molecule that otherwise primarily exhibits positive phase values (red). Since the phase is a 2π-periodic function, the change in sign in the phase can be attributed to continuous crossings of the +π/−π threshold (dark purple) (see also Figure S4a). The change in color thus corresponds to a continuous change in phase that can be related to structural features of the molecule: the blue spots within the molecule mark the regions of higher molecular thickness in the center of the subunits (Figure 3d).

The phase reconstruction of the alcohol dehydrogenase (ADH) molecule in Figure 3e features a region of increased phase shift values on the left side of the molecule. Due to the high symmetry of the molecule (Figure 3f), the molecule’s exact orientation cannot be unambiguously determined from the reconstructed molecular shape: several different orientations, which yield slightly different subunit arrangements, fit the observed molecular structure. The increased phase shift values in the left part of the molecule could thus be indicative of a structural feature that distinguishes this area from other parts of the molecule, suggesting an orientation as depicted in Figure 3f, in which the higher phase shifts correlate with the area featuring two overlapping subunits.

In all cases, the reconstructed phase distribution appears to trace the structural features present in the respective molecular models. The correlation is especially strong regarding variations in molecular thickness (in the direction of projection), which indicates that the reconstructed phase could be directly related to the number of atoms in the electron path and thus to the projected atomic density of the imaged molecules.

## Discussion

In high-energy electron imaging, the phase shift induced by the imaged object maps the mean inner potential of the object, which is defined as *V*_mean_ = , where *V*(*r*) is the electric potential of the object in a given volume Ω.^16,19^ If the potentials of the individual atoms within in the object do not differ strongly, this implies a direct connection between the induced phase shift and the density of the object or, in the case of a uniform density, the molecule’s thickness.

In imaging techniques employing low-energy electrons, the electron–electron nature of the interaction between beam and sample can lead to additional contributions to the phase shift, which complicates the interpretation of the measured phase data. In particular, exchange interaction,^19,42^ polarization effects,^42^ and inelastic scattering and absorption are not negligible in this energy range^19,42^ and could thus affect the observed phase shift. Still, the projected mean inner potential is likely to significantly contribute to the induced phase shift even at the low electron energies employed in LEEH.

In the case of proteins, which are primarily composed of atoms with similar scattering behavior, the projected mean inner potential can be approximated by the projected atomic density of the molecule, which serves as a measure of the number of atoms in the electron path, and can thus also be related to the thickness of the molecule, whose connection to the measured phase shift is supported by the examples presented in Figure 3. In the following, we underpin the relation between reconstructed phase shift and projected atomic density by comparing the phase reconstructions of two molecules of different thickness captured in the same hologram.

**Figure 4:**
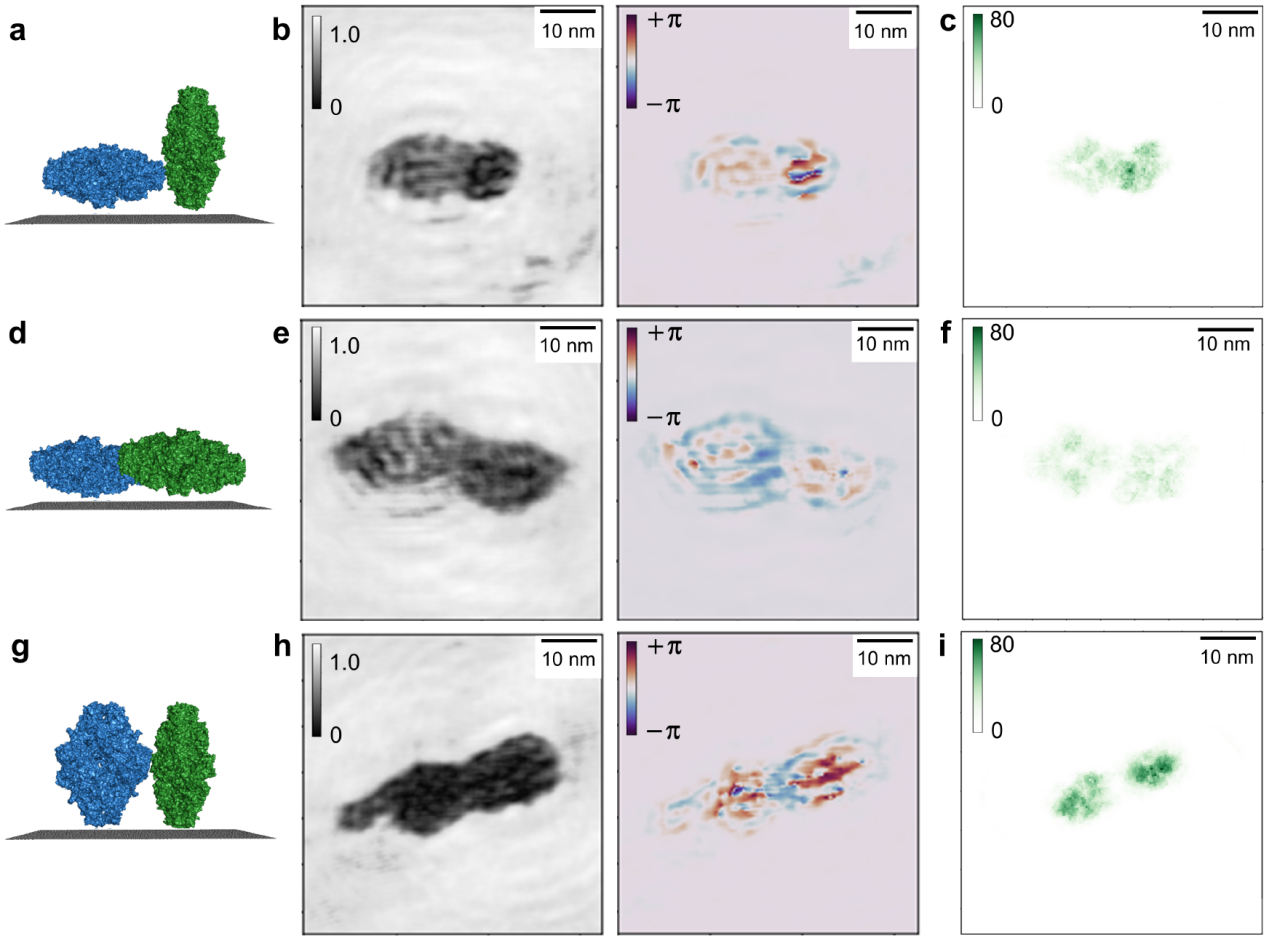
Correlation between reconstructed phase shift and projected atomic density. (a) Schematic of the orientation of the two β-galactosidase molecules in (b) with respect to the graphene substrate. The molecule on the left is in a flat orientation, whereas the molecule on the right is in an upright orientation; i.e., the molecules have different thicknesses as measured from the graphene. (b) Amplitude and phase reconstruction of a hologram featuring two β-galactosidase molecules in different orientations with respect to the substrate as schematically depicted in (a). (c) Projected atomic density of models of β-galactosidase (PDB: 6CVM) in orientations matching those in (b). Each pixel on the grid is colored according to the number of atoms projected into the pixel. The molecule in a flat orientation exhibits a lower overall projected atomic density than the molecule in upright orientation, which is reflected by the difference in overall phase shift generated by the two molecules. (d) Schematic of two β-galactosidase molecules in flat orientation. (e) Amplitude and phase reconstruction of a hologram featuring two β-galactosidase molecules in flat orientations with respect to the surface as sketched in (d). (f) Projected atomic density of the models corresponding to the orientations in (e). The overall projected atomic density as well as the overall phase shift of both molecules is similar. (g) Schematic of two β-galactosidase molecules in upright orientation. (h) Amplitude and phase reconstruction of a hologram featuring two β-galactosidase molecules in upright orientations with respect to the surface as sketched in (g). (i) Projected atomic density of the models corresponding to the orientations in (h). The overall projected atomic density as well as the overall phase shift of both molecules is similar, a slightly higher phase shift on the right can be correlated with an increased projected atomic density within the molecule on the right.

Figure 4 shows three such examples, each featuring two β-galactosidase molecules in different orientations with respect to the graphene substrate, which result in different thicknesses along the electron path. The respective orientations are sketched in the models in Figure 4a,d,g. Large proteins such as β-galactosidase are suitable for such an analysis since we expect that the contribution from the mean inner potential will dominate other effects, which may not be the case for smaller molecules. To be able to compare the reconstructions (Figure 4b,e,h) to the projected atomic density, projections of β-galactosidase models (PDB: 6CVM(43)) with orientations matching those of the reconstructed molecules are shown in Figure 4c,f,i. The projected atomic density plots are obtained by projecting the molecular models onto a grid, where each pixel (pixel size 4 Å × 4 Å) is colored according to the number of atoms projected onto it; i.e., darker regions correspond to a higher projected atomic density. To facilitate the comparison between all three examples, the plots have been scaled to the same value range.

**Figure 5:**
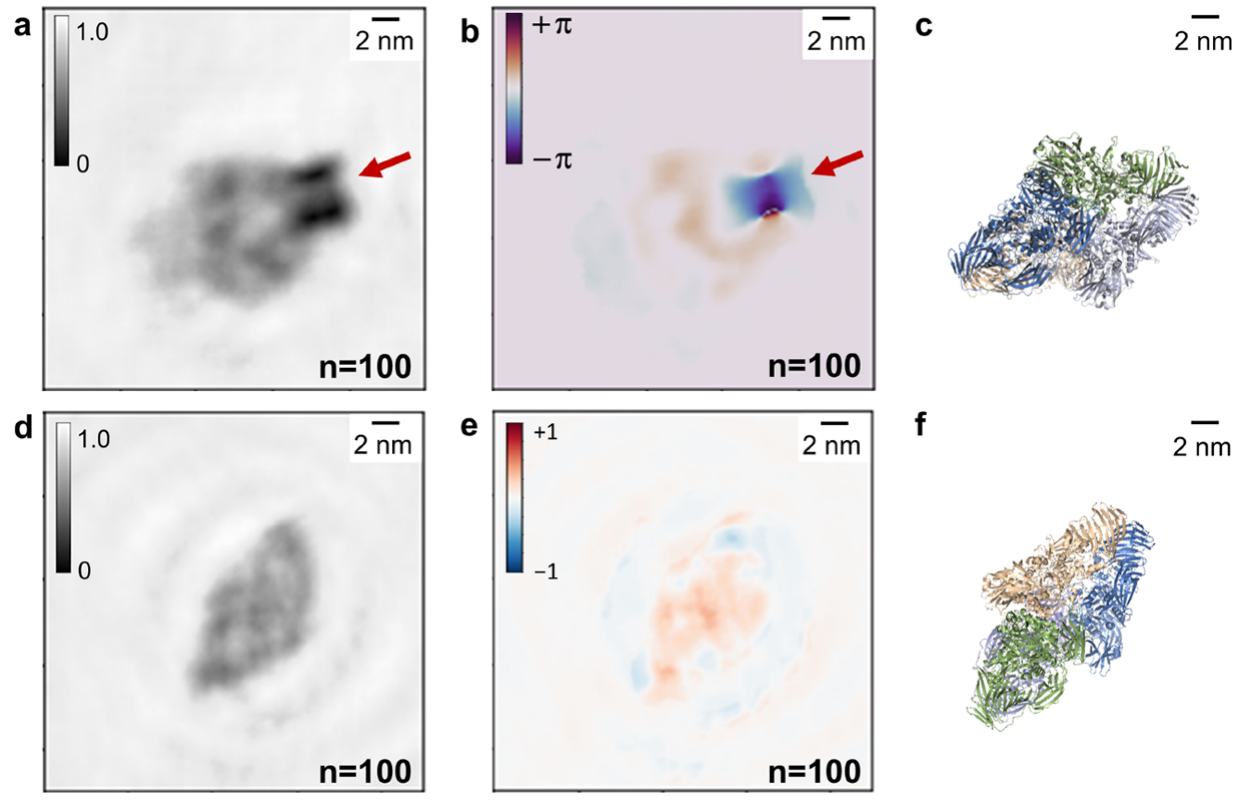
Sensitivity of the phase reconstruction to localized charges. (a, b) Iterative amplitude reconstruction (a) and iterative phase reconstruction (b) after 100 iterations of a hologram of a β-galactosidase molecule featuring a localized charge. The location of the charge is indicated by the red arrows. The charged feature is dominant, especially in the phase reconstruction. (c) Model of a β-galactosidase molecule (PDB: 6CVM(43)) in an orientation matching the one observed in the reconstructed images in (a, b). (d, e) Iterative amplitude reconstruction (d) and iterative phase reconstruction (e) after 100 iterations of a hologram of a β-galactosidase molecule in a similar orientation as the molecule in (a, b), but without a localized charged feature. Both amplitude and phase distribution feature values in a range similar to that observed in the protein signal in (a, b). (f) Model of a β-galactosidase molecule (PDB: 6CVM(43)) in an orientation matching the one observed in the reconstructed images in (d, e).

Figure 4a–c depicts a dimer consisting of two molecules in different orientations. As shown in Figure 4a, the molecule on the left is adsorbed in a flat orientation with respect to the surface; i.e., all four subunits composing the β-galactosidase molecule are in direct contact with the graphene. In contrast to this, the molecule on the right exhibits an upright orientation, where only part of the molecule is in contact with the substrate. This difference in orientation is reflected in the molecules’ projected atomic densities: the molecule on the left has a lower mean projected atomic density value of approximately 30 atoms per pixel and features large areas of low projected atomic density, while the molecule on the right has a higher mean projected atomic density (approximately 50 atoms per pixel) as well as a higher maximum projected atomic density value of 75 atoms per pixel (Figure 4c). The differences in projected atomic density are in turn reproduced in the phase reconstruction: while there are internal phase variations in both molecules, the overall phase shift induced by the molecule on the left (approximately 0.5 rad) is much lower than the overall phase shift (approximately 1–2 rad, with locally higher values close to π rad) induced by the molecule on the right. The internal variations observed in both the reconstructed amplitude and phase distributions cannot be interpreted unequivocally. They could map internal structural details of the molecules, as for example, in the case of the molecules depicted in Figure 3 and Figure 5a. Given the periodicity of the features in Figure 4, however, they could also be artifacts caused by modulations in the hologram created by the fringes of other objects in the proximity of the molecules of interest.

In Figure 4d–f, two β-galactosidase molecules are depicted which are both adsorbed in flat orientations, resulting in similar projected atomic density distributions (Figure 4f, mean projected atomic density 10–20 atoms per pixel). The phase reconstruction reflects this; the phase shifts induced by both molecules are indeed almost identical (approximately 0.5 rad, Figure 4e).

Finally, Figure 4g–i features two molecules that can both be associated with upright orientations with respect to the graphene surface. The differences in size and shape in comparison to the upright molecule in Figure 4b suggest that these molecules are not fully upright, but in an intermediate orientation that is neither fully flat nor fully upright. While the overall projected atomic densities (approximately 50–60 atoms per pixel) and phase shifts (approximately 1.5 rad on the left, approximately 2 rad on the right) are similar in both molecules (Figure 4h,i), the molecule on the right features a higher maximum projected atomic density (80 atoms per pixel), which correlates with the slightly increased phase shift observed in the right molecule.

The analysis of all three cases suggests that the phase shift induced by a molecule is indeed related to the projected atomic density and thus to the projected mean inner potential of the molecule. An accurate quantitative comparison between the reconstructed phase shift and theoretically obtained phase shift values is, however, difficult to attain. A precise theoretical estimate would have to be derived from the electrostatic protein potential, which is inaccessible for such highly complex molecules given the current computational limitations. However, assuming that the mean inner potential is the principal contribution to the reconstructed phase shift, the expected total phase shift can be estimated by calculating the phase shift induced by light atoms (C, N, O), which are the main elements composing a protein, and summing the resulting phase contributions according to the projected atomic density. Using a partial wave-based scattering algorithm for hologram simulation,^44^ the phase shifts of individual carbon, nitrogen, or oxygen atoms are found to be in the range of 0.05 rad. For a β-galactosidase molecule in a flat orientation (projected atomic density approximately 10–30 atoms per pixel) one would hence expect a phase shift in the range of 0.5–1.5 rad. For the molecules in upright orientations, the estimated phase shift is 2–4 rad (projected atomic density approximately 40–80 atoms per pixel). In both cases, the estimated phase shifts are in the same range as the experimentally reconstructed values. While we observe a correlation between measured phase shift and projected atomic density as well as a degree of quantitative agreement between the estimated and observed phase shifts, both the phase shift variations within the molecule and potential further contributions to the induced phase shift at the low electron energies employed in the experiment complicate the interpretation of LEEH phase data. For a more precise quantitative description of the interrelation between phase shift and projected atomic density, further data at a larger variety of different projected atomic densities or molecular thicknesses is required.

Given the sensitivity of the reconstructed phase shift to local changes in the projected mean inner potential of the molecule, strong phase signatures would be expected for larger changes in electron density, which could, for example, be related to strong differences in scattering behavior or to localized net charges on or close to the molecule.

As reported in previous studies,^23,45^ charged defects or adsorbates can be observed on graphene by LEEH imaging. Such features are also reported to produce clear signatures in the reconstructed phase shift, suggesting a direct relation between the phase shift and the electric potential of charged adsorbates or defects on graphene surfaces. It is hence of interest to examine the influence of the electrostatic potential created by localized charges on the phase reconstruction of protein holograms. Figure 5a,b shows the reconstruction of a β-galactosidase molecule landed on a negatively charged feature on the graphene substrate, which can be identified by a comparison of the sample before and after deposition of the proteins and has an estimated charge of 4–5 electron charges (see Figure S5). The localized charge appears as a dark spot in the amplitude reconstruction (Figure 5a) and as a high-contrast negative-phase feature in the corresponding phase reconstruction (Figure 5b), as indicated by the red arrows.

In the phase reconstruction, the localized charge generates a higher phase shift than the protein itself. In order to substantiate this observation, the reconstructed amplitude and phase of a different β-galactosidase molecule without a localized charge, landed in a similar orientation as the one in Figure 5a,b, is reported for comparison in Figure 5d,e. In both cases, the phase shift induced by the presence of the molecules is comparable (approximately 0.5 rad). The dominant phase signature in Figure 5b can thus be attributed to the localized charge. These findings demonstrate that the reconstructed phase shift is sensitive to the increased potential created by a localized charge, which further supports the interpretation that the phase reconstruction maps local changes of potential within the imaged protein molecules.

In addition, the molecule-charge system shown in Figure 5a,b provides an example of the circumstances in which both positive and negative phase shifts can be expected in the same reconstruction. This can occur when charges of different signs, which would be expected to yield phase shifts of opposite sign, accumulate in different parts of the molecule or, as in the present example, if the detected phase shifts can be traced to different origins, such as the mean inner potential of the protein and the electrostatic potential of a charged feature. The high sensitivity of low-energy electrons to small changes in local potential and charge distribution could thus allow the mapping and interpretation of phase shift distributions induced by biomolecules.

## Conclusion

We have shown that it is possible to retrieve amplitude and phase distributions from experimentally acquired LEEH holograms of individual proteins by applying an iterative phase retrieval algorithm. The constraints, based on those reported in Latychevskaia et al.,^14^ allow for a robust iterative phase reconstruction of both simulated and experimentally acquired holograms, indicating that this approach could be extended to different classes of molecules beyond proteins. The effective suppression of the twin image contributions in the reconstructed images yields a clearer visualization of the molecular shape without ambiguities due to artifacts. Beyond improving the quality of the reconstructed images, we have demonstrated that the reconstructed phase images provide information about the local potential interacting with the incident electrons. This, on the one hand, emphasizes the possibility of mapping localized electric fields by phase imaging; on the other hand, it yields insights about the mean inner potential of the molecule, as evidenced by the strong correlation between the measured phase shift distribution and the projected atomic density, i.e., the thickness, of the imaged molecules.

The iterative reconstruction method thus shows a route toward identifying structural features related to local changes in potential within the imaged molecules. Furthermore, at high spatial resolution, the ability to identify and map localized potentials could be used to extract chemical information from holograms.^44^ However, because of the complex interaction of low-energy electrons with biological matter, further contributions likely need to be taken into account in the interpretation of the reconstructed phase maps. This could be elucidated by a comparison of the experimental phase data to simulations generated from quantitatively accurate potentials describing the investigated molecules.

## Methods

### Low-Energy Electron Holography

The LEEH microscope used for the experimental acquisition of the protein holograms presented here is set up in an in-line holography geometry;^10^ i.e., the electron source, the sample, and the detector are located along the same optical axis. The electron source is a sharp tungsten tip, which produces low-energy electrons (50–200 eV) when brought in close distance (approximately 200–500 nm) to the SLG substrate, which is a distance suitable for the imaging of individual protein molecules. No lenses or other optical elements are used. The tuning of the tip–sample distance allows a change in magnification, permitting the acquisition of both survey images (Figure S5) and holograms of individual molecules.

Typical hologram acquisition times are in the millisecond range and typical doses are in the range of 10^6^ e^–^ per nm^2^. The LEEH microscope operates at a base pressure of 10^–10^ mbar and under room temperature conditions. The tungsten emitters are prepared by electrochemical etching in a 20 wt % NaOH solution, followed by self-sputtering and annealing procedures in UHV.

Because an undistorted reference wave is crucial for LEEH imaging, an atomically clean, electron-transparent substrate is required. Since SLG is transparent to electrons in the energy range at which LEEH operates, a clean graphene substrate can be assumed to only induce a global phase shift, thus allowing the reference wave to remain undisturbed during the propagation to the detector. Hence, the relative background phase shift given by the phase of the reference wave can be considered constant and can thus be set to zero. Furthermore, graphene is conductive, which reduces charging effects, thus allowing for distortion-free imaging, and only interacts weakly with biomolecules, thereby minimizing structural changes to the imaged objects due to interactions with the substrate.^36^

### Sample Preparation

Since LEEH imaging requires ultraclean sample conditions, the proteins are deposited on SLG by native ES-IBD,^35^ which allows for a clean and controlled sample preparation while ensuring that the deposited molecules remain chemically intact by employing mass spectrometry and mass selection methods before deposition.^3,4,35,46^ To retain a native state of the proteins in the spray solution, 200 mM ammonium acetate is used as solvent and low-flow pulled glass capillary tips, operated at low spray voltages (in the range of 1–1.5 kV), are utilized. The concentration of the spray solutions was 0.5 mg/mL. The protein deposition is carried out in an ultrahigh vacuum (UHV, *p* ≈ 10^–10^ mbar) environment using soft landing conditions (kinetic energy upon landing below 5 eV per charge).

The SLG substrates are prepared according to the procedure described in Longchamp et al.,^47^ and the purity of the SLG is ascertained by LEEH before the deposition of the molecules.

### Image Processing

When performing one-step amplitude reconstructions of experimentally acquired proteins,^3^ the photograph of the hologram displayed on the detector can directly be used as input for the reconstruction algorithm without any further image processing steps. For the iterative reconstruction, image preprocessing is necessary to avoid artifacts in the reconstruction. The processing steps encompass the cropping of the hologram such that only the hologram of the molecule of interest remains, a polynomial background subtraction resulting in a uniform background to avoid edge artifacts and filtering in the Fourier domain to reduce high-frequency noise in the hologram, which enhances the fringe pattern. To further suppress potential edge artifacts, an apodization filter can be used as a final processing step. Additionally, the processed holograms are normalized by division by the mean value of the hologram intensity.

## Supporting Information Available

Full description of the iterative reconstruction algorithm; convergence of the iterative reconstruction algorithm; relevance of the phase constraint, robustness of the algorithm regarding different initial phase distributions; relevance of the phase constraint, robustness of the algorithm regarding high absorption values; unwrapping phase data; comparison of phase unwrapping algorithms; origin of the localized charge in Figure 5a,b (PDF)
