# Supplementary material for: Phase Reconstruction of Low-Energy Electron Holograms of Individual Proteins
Source: ACS Nano. 2022 Nov 11;16(11):18568–78. doi: 10.1021/acsnano.2c06897 (PMC9706659; doi:10.1021/acsnano.2c06897)
Supplement: Supplementary file 1 — nn2c06897_si_001.pdf [file nn2c06897_si_001.pdf]

Supporting Information for "Phase reconstruction of low-energy electron holograms of individual proteins"

Hannah Ochner[1,*], Sven Szilagyi[1], Moritz Edte[1], Luigi Malavolti[1,*], Stephan Rauschenbach[1,2], and Klaus Kern[1,3]

[1]Max Planck Institute for Solid State Research, Heisenbergstr. 1, DE-70569 Stuttgart, Germany

[2]Department of Chemistry, University of Oxford, 12 Mansfield Road, Oxford OX1 3TA, UK

[3]Institut de Physique, École Polytechnique Fédérale de Lausanne, 1015 Lausanne, Switzerland

[*]Corresponding authors: Hannah Ochner (h.ochner@fkf.mpg.de), Luigi Malavolti (l.malavolti@fkf.mpg.de)

Full description of the iterative reconstruction algorithm

The iterative reconstruction algorithm discussed in this paper was first described in [1] and takes the following form (see also Fig. 1b, main text):

As a first step, the complex wave field at the detector is formed from the initial input, consisting of the measured amplitude distribution in the hologram plane, $A = \sqrt{H}$, i. e. the square root of the measured hologram H, and an initial phase distribution ϕ_{init}, which can be chosen randomly. The resulting complex wave field at the detector takes the form $U = Ae^{i\phi_{init}}$. Subsequently, the complex wave field U is propagated from the hologram plane to the object plane by Fresnel-Kirchhoff propagation, yielding a first estimate of the transmission function of the object (iteration step n = 0), $t = ae^{i\varphi}$, with amplitude distribution a and phase distribution φ. As a next step, the constraints in the object plane are enforced, resulting in a modified transmission function $t' = a'e^{i\varphi'}$.

The constraints applied on the object level require the absorption and phase values of all pixels with negative absorption values (*i.e.* amplitude > 1) to be set to 0. Following that, the new transmission function t' is propagated to the hologram plane, yielding a new complex wave field $U' = A'e^{i\Phi'}$. The constraint in the hologram plane is applied, *i.e.* the calculated amplitude distribution A' is replaced by the measured amplitude distribution $A = \sqrt{H}$. The resulting complex wave field $U = Ae^{i\Phi'}$ is used as the input for the next iteration of the algorithm.

Convergence of the iterative reconstruction algorithm

The convergence of the iterative algorithm can be quantified by calculating the error between reconstructions obtained from subsequent iteration steps. For this, the mean squared error can be used:

$$E_A = \frac{1}{N^2}\sum_{i,j=0}^{N} |a_{n-1}(i,j) - a_n(i,j)|^2 \qquad (1)$$

$$E_\phi = \frac{1}{N^2}\sum_{i,j=0}^{N} ||\varphi_{n-1}(i,j)| - |\varphi_n(i,j)||^2 \qquad (2),$$

where E_A is the error between consecutive reconstructed amplitude images, E_Φ is the error between consecutive reconstructed phase images, N^2 is the number of pixels in the images and a_n and φ_n are the amplitude and phase reconstructions obtained in the nth iteration step. The summation is carried out over all pixels *(i,j)*. Convergence is reached when the errors reach certain threshold values and do not decrease further (see Figure S3). In the experimental case, this threshold value is in the range of 10^{-5} to 10^{-6}, in the case of simulated objects, the error is below 10^{-7}. Adding noise to simulated images yields errors in a similar range as in the experimental case. Since reconstructions of experimentally acquired holograms usually converge in less than 50 iterations, and the error, while fluctuating slightly, remains in the same range after the threshold value is reached (see Figure S3), we use 100 iterations as a default to have a standard for comparison. As shown in Figure S1, the algorithm converges robustly to the same result for different initial phase inputs.

For simulated objects, the error between the reconstructed image and the input can be calculated in a similar way:

$$E_a = \frac{1}{N^2}\sum_{i,j=0}^{N} |a_{in}(i,j) - a_{rec}(i,j)|^2$$

$$E_\varphi = \frac{1}{N^2}\sum_{i,j=0}^{N} ||\varphi_{in}(i,j)| - |\varphi_{rec}(i,j)||^2$$

where a_{in} and φ_{in} denote the input amplitude and phase distributions and a_{rec} and φ_{rec} denote the reconstructed amplitude and phase distributions. The errors obtained between input and reconstruction are similar to the errors calculated between subsequent iterations once convergence is reached.

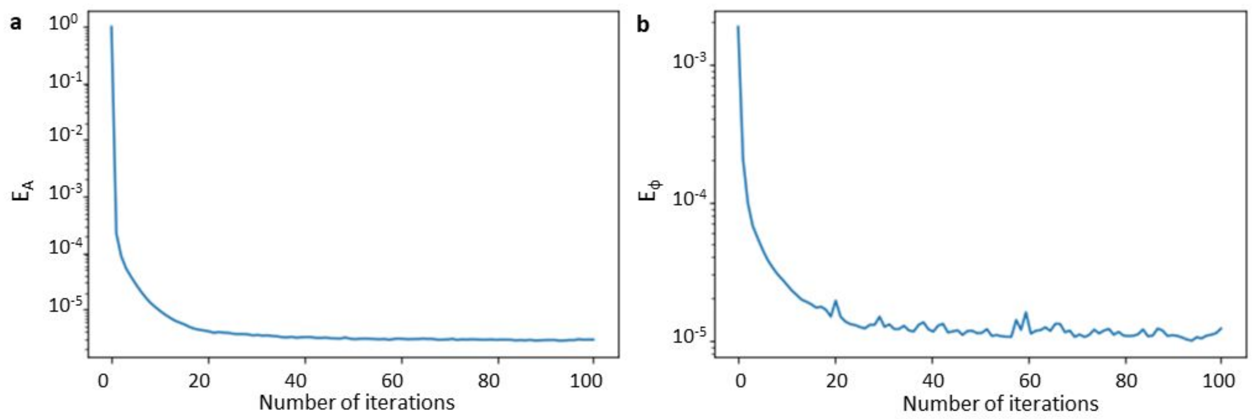

Figure S1: Convergence of the iterative reconstruction algorithm. a Plot of the mean squared error of subsequent amplitude reconstructions E_A as defined in equation (1) vs. the number of iterations for an experimentally acquired hologram of a hemoglobin molecule. **b** Plot of the mean squared error of subsequent phase reconstructions E_ϕ as defined in equation (2) vs. the number of iterations for the same hologram as in **a**.

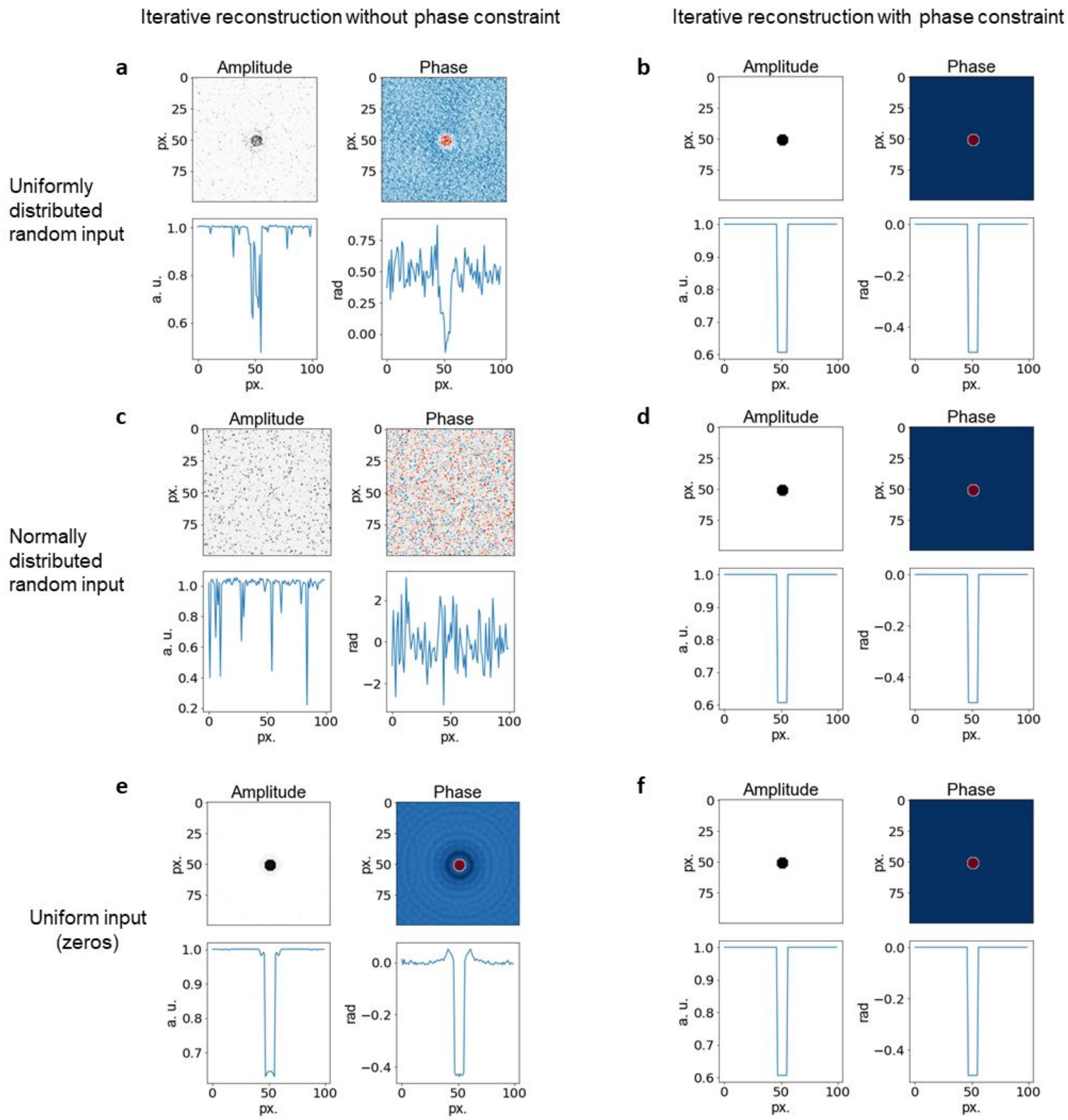

Figure S2: Relevance of the phase constraint: Robustness of the algorithm regarding different initial phase distributions. Iterative reconstruction of a hologram of a disk with amplitude a=0.65 a.u. and phase φ=-0.5 rad with (**b**, **d**, **f**) and without (**a**, **c**, **e**) the application of the phase constraint described in the main text for different initial phase distributions: a uniform distribution of random numbers generated by the NumPy function `rand()` (**a**, **b**), a normal distribution of random numbers generated by the NumPy function `randn()` (**c**, **d**), and a uniform input distribution consisting of an array of zeros (**e**, **f**). While the iterative reconstruction with phase constraint retrieves the input independently of the initial phase distribution (**b**, **d**, **f**), the result of the reconstruction without phase constraint depends strongly on the choice of initial input (**a**, **c**, **e**).

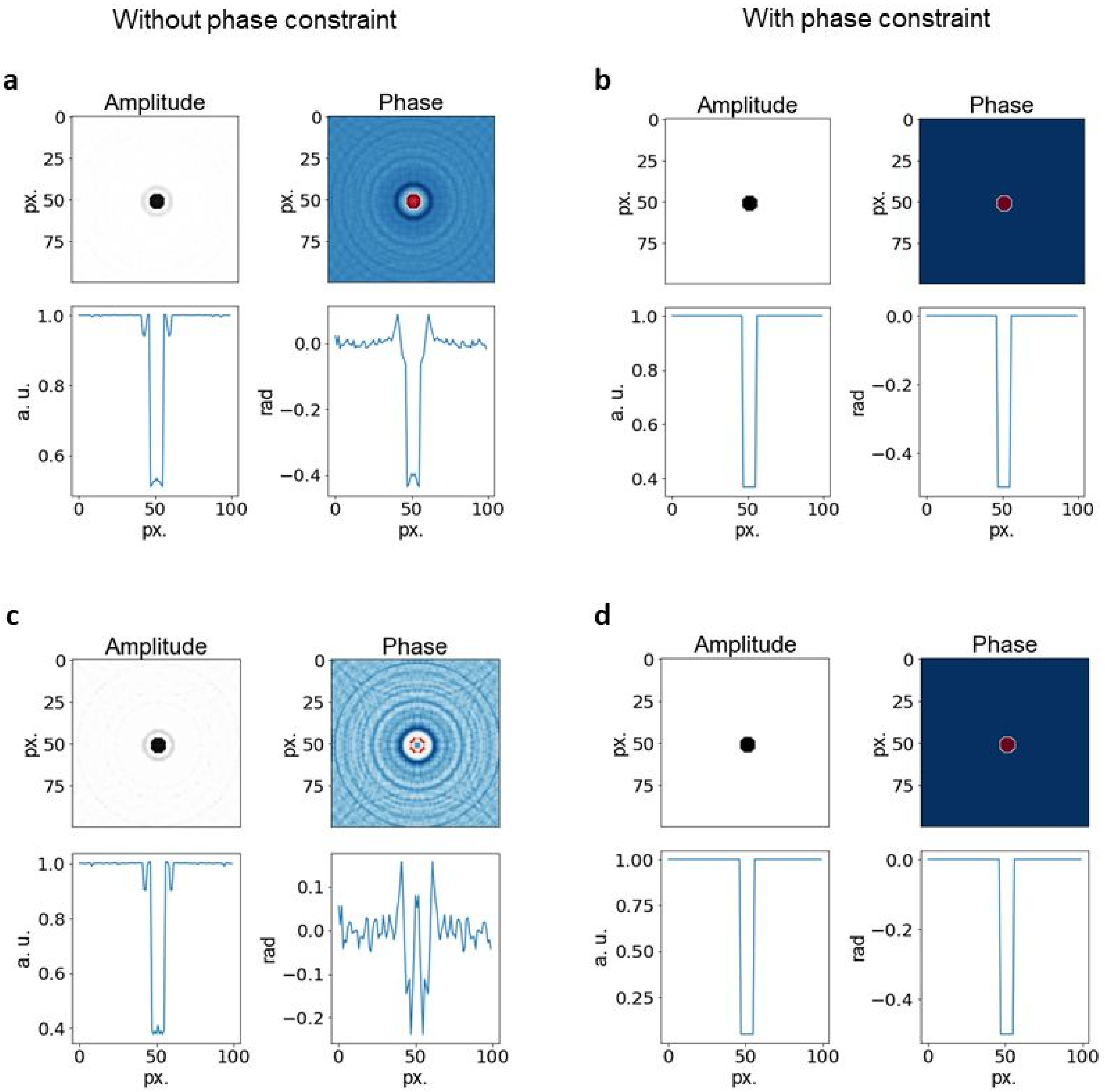

Figure S3: Relevance of the phase constraint: Robustness of the algorithm regarding high absorption values. a, **b**: Iterative reconstruction of a disk of absorption α=1 a.u. (amplitude a=0.37 a.u.) and phase φ=-0.5 rad with (**b**) and without (**a**) phase constraint. **c**, **d**: Iterative reconstruction of a disk of absorption α=3 a.u. (amplitude a=0.05 a.u.) and phase φ=-0.5 rad with (**d**) and without (**c**) phase constraint. The reconstruction with phase constraint (**b**, **d**) retrieves the input independently of the choice of absorption value, the reconstruction without phase constraint deteriorates with increasing absorption (**a**, **c**). Since especially larger proteins can be expected to have strong absorbing properties, this underlines the importance of constraining the phase along with the amplitude.
The holograms used in Figures S1 and S2 have been simulated from a complex-valued transmission function using a Fresnel-Kirchhoff propagation-based process as detailed in [2].

Unwrapping phase data

The primary output of the iterative reconstruction algorithm is a phase map projected on the interval [-π, π). In cases in which the maximum (minimum) value of the reconstructed phase is π (-π), the phase can be considered as wrapped, *i.e.* the phases are correct relative to one another, but phase shifts larger than π are projected back onto the interval [-π, π). In principle, it is possible to try to retrieve the absolute phase values by employing phase unwrapping algorithms. However, phase unwrapping of two-dimensional images is not a straightforward process and many different algorithms have been proposed. In Figure S4, we have applied two different phase unwrapping algorithms to experimental LEEH data, the one proposed by Herráez et al. in [3] as implemented in the `skimage.restoration` function `unwrap_phase` and the algorithm outlined by Schofield and Zhu in [4]. As can be seen from comparing the results of the unwrapping operations, the unwrapped phase distributions recovered using the different algorithms differ both in the retrieved absolute phase values and in their convergence properties (see Figure S4).

In the transferrin example presented in Fig. S4a, the algorithm by Schofield and Zhu produces the qualitatively expected unwrapped distribution, converting the areas of negative phase within the molecule into areas of high positive phase. For the same hologram, the `skimage.restoration` function does not fully achieve such a result, although the π/-π crossings that appear as a colour change from dark red to dark blue in the wrapped phase are resolved in a similar way (green box in Figure S4a). In other cases, there are phase features which neither of the algorithms manages to fully resolve, *e.g*. the change from positive to negative phase within the ADH molecule in Figure S4b.

Comparing the performance of the two algorithms more generally, both on simulated and experimental examples, we found that while the algorithm by Schofield and Zhu has better convergence properties, the `skimage.restoration` implementation of the algorithm by Herráez et al. performs much better on simulated data, especially in cases featuring larger phase differences. This comparison shows that it is not straightforward to choose a suitable phase unwrapping algorithm since they can yield quantitatively different results and may even introduce further artefacts. Given that, we chose to show the direct output of our iterative reconstruction scheme in the main text.

The example in Figure S4c shows that in all three cases the phase feature associated with the localised charge yields a negative phase shift (all three algorithms also agree quantitatively). This supports the conclusion that the change in phase sign observed here is not an artefact of the algorithm, but indeed a physical feature originating from the repulsive potential of the negative charge (negative phase shift) that is in close proximity to protein, which creates an overall positive phase shift (see also main text and Fig. S5).

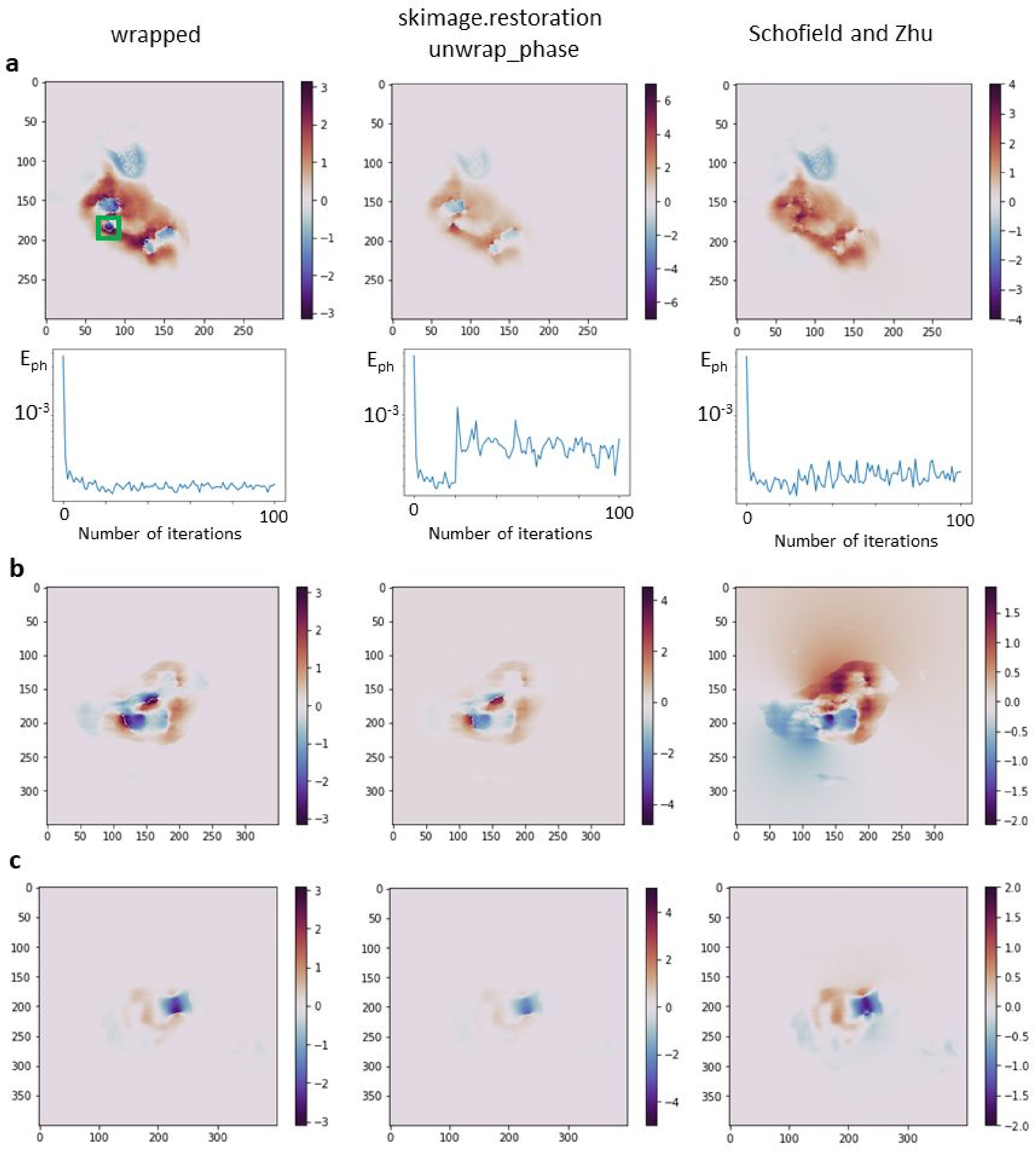

Figure S4. Comparison of phase unwrapping algorithms. Comparison of the wrapped phase (left), the unwrapped phase distribution provided by the `skimage.restoration` function `unwrap_phase` (centre) and the unwrapped phase distribution provided by an unwrapping algorithm based on the suggestion by Schofield and Zhu (right) applied to three different holograms of proteins that are also discussed in the main text: transferrin (**a**), ADH (**b**) and β-Galactosidase (**c**). In **a**, the convergence properties of each of the algorithms are also shown.

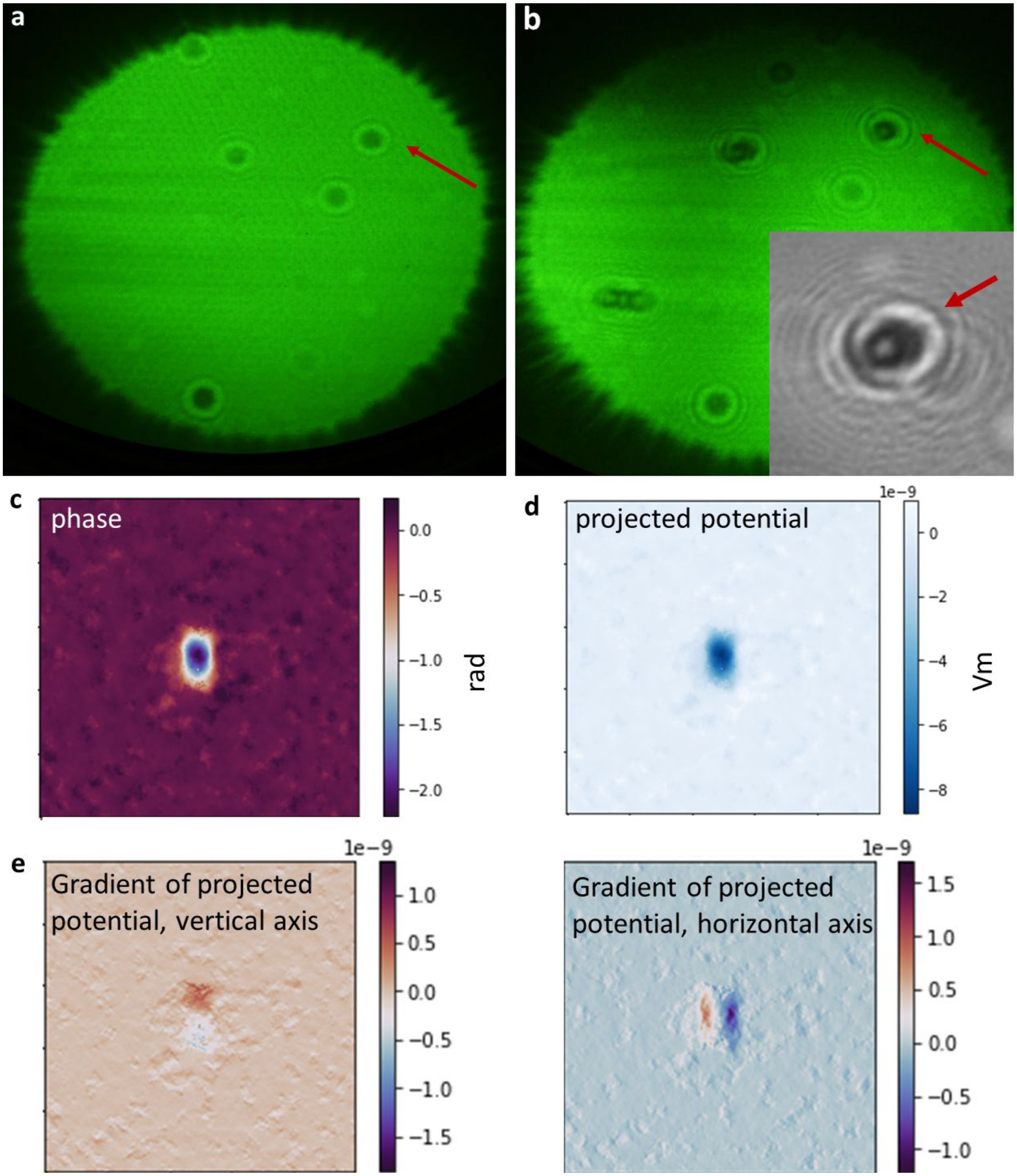

Figure S5: Origin of the localised charge in Fig. 5a, b. a Survey hologram of the single-layer graphene substrate before the deposition of the molecules. A charged feature is already present in the location where the molecule is observed after deposition (red arrow). The dark centre of the feature in the hologram indicates that it is negatively charged [5]. **b** Survey hologram of the graphene substrate after

deposition, indicating that a has molecule adsorbed at the location in question (red arrow). The inset shows the processed hologram from which the reconstructions in Fig. 5a, b are obtained. The contribution of the charge is visible in the asymmetry of the hologram pointed out by the arrow. **c** Phase reconstruction of the charge from the hologram shown in **a**. Inverting the formula given in [5] for calculating the phase shift of a charge q, the reconstructed phase shift suggests that the charge present here is in the range of 4-5 electron charges, which is a reasonable approach since the reconstructed phase values do not exceed π. **d** Projected potential corresponding to the phase distribution in **c** as calculated by equation (6) in [6]. **e** Negative gradient of the projected potential in **d** along the vertical and horizontal directions, which can be used as an estimate for the corresponding electric field.

References

[1] Latychevskaia, T.; Fink, H.-W. Solution to the Twin Image Problem in Holography. *Phys. Rev. Lett.* **2007**, *98*, 233901.

[2] Latychevskaia, T.; Fink, H.-W. Practical algorithms for simulation and reconstruction of digital in-line holograms. *Appl. Opt.*, 2015, 54, 2424.

[3] Herráez, M. A. ; Burton, D. R. ; Lalor, M. J.; Gdeisat, M. A. Fast Two-Dimensional Phase-Unwrapping Algorithm Based on Sorting by Reliability Following a Noncontinuous Path. *Appl. Opt.*, 2002, 41, pp. 7437-7444.

[4] Schofield, M. A.: Zhu, Y. Fast Phase Unwrapping for Interoferometric Applications. Opt. Lett., 2003, 28, 1194-1196.

[5] Latychevskaia, T.; Wicki, F.; Longchamp, J.-N.; Escher, C.; Fink, H.-W. Direct Observation of Individual Charges and Their Dynamics on Graphene by Low-Energy Electron Holography. *Nano Lett.* 2016, 16, 9, 5469–5474.

[6] Latychevskaia, T.; Wicki, F.; Escher, C.; Fink, H. W. Imaging the Potential Distribution of Individual Charged Impurities on Graphene by Low-Energy Electron Holography. *Ultramicroscopy* **2017**, *182*, 276–282.
